# Family Members’ Experiences in a Psychiatric Hospital During the COVID-19 Pandemic: Implications for Family-Centered Work in Mental Health Systems

**DOI:** 10.1192/j.eurpsy.2023.1247

**Published:** 2023-07-19

**Authors:** R. Shor, A. Shalev

**Affiliations:** 1 School of Social Work and Social Welfare, The Hebrew University of Jerusalem, Jerusalem; 2 Faculty of Social Work, Ashkelon Academic College, Ashkelon, Israel

## Abstract

**Introduction:**

Restricted visitations of family members of persons with mental illness in psychiatric hospitals which may occur during times of public health crises such as the COVID-19 pandemic can have potential adverse consequences on the family members and on their ability to fulfill the caregiving role. Therefore, mental health professionals may encounter difficulties implementing a family centered-care model during such periods.

**Objectives:**

Due to the limited knowledge about the effects of the restrictions during the pandemic a study was conducted in Israel. It examined the difficulties which family members experienced as a result of the restricted visitations and the effects of the restrictions on advancing a family-centered care model,

**Methods:**

A semi-structured questionnaire was distributed via digital means to 75 family members who had a family member who was hospitalized in psychiatric hospitals during the pandemic.

**Results:**

The findings indicate that family members had limited opportunities in the following areas during the COVID-19 area: Developing relationships with the professional staff, being involved in the therapeutic process during the hospitalization, communicating with the hospitalized family member, and receiving help for themselves.

**Conclusions:**

Mental health professionals in psychiatric hospitals should adapt family-centric procedures to circumvent restrictions on physical presence and maintain the involvement of family members during psychiatric hospitalization.

**Disclosure of Interest:**

None Declared

